# Social distancing and mental health among pregnant women during the coronavirus pandemic

**DOI:** 10.1186/s12905-023-02335-x

**Published:** 2023-04-20

**Authors:** Emily W. Harville, Moira E. Wood, Elizabeth F. Sutton

**Affiliations:** 1 Department of Epidemiology, Tulane School of Public Health and Tropical Medicine, 1440 Canal St Ste 2001 #8318, New Orleans, LA 70112-2715 USA; 2 Tulane University School of Medicine, New Orleans, LA USA; 3 Woman’s Hospital Research Institute, Baton Rouge, LA USA

**Keywords:** Social distancing, COVID-19 pandemic, Anxiety, Pregnancy, Postpartum

## Abstract

**Background:**

The effect of social distancing due to the COVID-19 pandemic on the mental health of pregnant women is of particular concern, given potential effects on physical health, family functioning, and child development.

**Methods:**

Pregnant women were recruited for the “Implications of and Experiences Surrounding being Pregnant during the COVID-19 Pandemic” study at Woman’s Hospital in Baton Rouge, Louisiana. Participants enrolled at any point during their pregnancy and surveys were delivered weekly until the participant indicated that she had delivered her baby; a postpartum survey followed four weeks after delivery. This analysis includes 1037 participants with baseline, 596 with follow-up, and 302 with postpartum surveys. Questions on social distancing behaviors were asked at baseline and grouped based on whether they involved social distancing from work, friends and family, or public places. Symptoms of anxiety, stress, depression, and pregnancy-related anxiety were measured. Each type of social distancing was examined as a predictor of mental health using linear model with control for confounders.

**Results:**

The study population was largely white (84.1%), married (81.8%), and educated (76.2% with a bachelor’s or higher degree). Women who were younger, Black, unmarried, or had less education or income reported fewer social distancing behaviors. Mean anxiety score in the highest quartile of overall social distancing was 8.3 (SD 5.6), while in the lowest quartile it was 6.0 (SD 5.0) (*p* < 0.01), while perceived stress postpartum and pregnancy-related stress were not associated with social distancing. Associations were substantially diminished when controlled for baseline levels of anxiety symptoms.

**Conclusions:**

Greater social distancing was associated with more anxiety symptoms, but worse mental health, particularly anxiety, may also have contributed to greater social distancing behaviors.

**Supplementary Information:**

The online version contains supplementary material available at 10.1186/s12905-023-02335-x.

## Background

The mental health of pregnant women may have been particularly affected by the COVID-19 pandemic [1] and symptoms of depression, anxiety, stress, and post-traumatic stress disorder (PTSD) have increased among pregnant women during the pandemic [2]. The combination of the usual concerns of pregnancy and the population-wide stressors of the pandemic created particular hardship. Beyond the increased levels of depression and anxiety being reported as a direct result of being pregnant during a pandemic, pregnancy brought associated stressors related to child well-being and access to childcare and medical care [3, 4]. In a national (U.S.) survey, perceived pregnancy-related anxiety was related to factors directly affected by the pandemic, such as stopping face-to-face prenatal visits and changing birth plans, as well as fear of running out of food, increased conflict in the home, and fear of getting infected with COVID-19 [5]. Other studies point to pregnancy- and pandemic-specific stressors such as worry about having their partner with them at the birth as drivers of adverse mental health [6]. An analysis across multiple high-income countries implicated both pregnancy-specific and pandemic-related pregnancy stress as drivers of depression and anxiety among pregnant women [7].

Perceived social support can lessen psychological symptoms [8]. Perceived stress and lack of social support are fundamental effectors of prenatal anxiety, and social support is the most powerful coping mechanism to mediate stressful situations, including pregnancy [9]. Limited social support is associated with increased risk of depression and anxiety during pregnancy [10]. This breakdown in the networks that pregnant women used to seek comfort in may foster extreme feelings of isolation and depression during the COVID-19 pandemic [11]. When the COVID-19 pandemic forced people inside and online, many of the ways people found social support were no longer viable options [12]. Stay-at-home orders, virtual work, closed schools, and limits on both public and private gatherings yielded a reality where most social avenues were closed. Previous outbreaks and pandemics show the deleterious effects of social isolation [13, 14], and such isolating behaviors have been intensely stressful for many people. However, evidence is contradictory: at least one study suggests that more stringent government containment measures were associated with better mental health among pregnant women [15], while others find higher COVID-19 restrictions to be associated with more stress and worse mental health [16]. In this study, we examined how various types of social distancing were associated with mental health and stress in a sample of pregnant women. This population is of acute concern given that maternal mental health is tied to pregnancy complications, birth outcomes, infant health, and child development, and is sometimes found to be at higher risk of stress-related adverse mental health [3], including during the pandemic [16]. We hypothesized that women who adhered to more social distancing behaviors would be more likely to report symptoms of poor mental health (anxiety, stress, and depression).

## Methods

### Study design and recruitment

Pregnant women were recruited for the “Implications of and Experiences Surrounding being Pregnant during the COVID-19 Pandemic” at Woman’s Hospital in Baton Rouge, Louisiana. Participants were recruited via advertisements communicated through participating institutions’ outreach resources (hospital newsletter, the Woman’s Hospital Pregnancy app, social media, and the Woman’s Hospital obstetrical nursing hotline) and via targeted, secure notifications (MyChart messages, text messages) when permissible by institutional policies. If participants began the survey or indicated interest to their prenatal care provider but did not complete the survey, a research assistant contacted them and encouraged them to complete the survey. Because recruitment was virtual, receiving care at a participating hospital was not a requirement, but a large majority of the participants received care on site.

This analysis covers up to 3 surveys. Participants enrolled in the study at any point during their pregnancy and completed the baseline survey at this point. The first follow-up survey was sent one week after baseline; if not completed then, reminders continued to be sent until they completed it or delivered their baby; median time of completion was 1.6 weeks after baseline. An additional survey was sent 4 weeks postpartum. Woman’s Hospital limited visitors starting on March 13, 2020, and Louisiana issued a stay-at-home order on March 22, 2020. Recruitment began in April 2020 at Woman’s Hospital. Louisiana entered phase 1 of lifting of requirements (stay at home order removed, occupancy restrictions, masks and social distancing required) in May 2020, phase 2 (more businesses open) in June 2020, phase 3 in September 2020, and returned to phase 2 in November 2020. In March of 2021, the state returned to phase 3 [17]. This analysis includes participation through March 2021: 1037 participants with baseline surveys; 596 with follow-up surveys; and 302 with postpartum surveys (Table 1). Participants were sent the postpartum survey regardless of whether they had completed follow-up surveys previously.

**Table 1:** Timing and sample size of measures

	Baseline	Follow-up	Postpartum	N with baseline and follow-up time point	N with three time points
Anxiety	1037	595	302	572	208
Depression		595	301	253	
Perceived stress	919		301	286	
Pregnancy-related stress	765				
Pregnancy-related anxiety		596		552^a^	

^a^N with baseline general anxiety

### Social distancing behaviors

Questions on social distancing behaviors were asked at baseline about behavior over the last 7 days. We created scales of social distancing behaviors based on whether they involved social distancing from work, friends and family, or public places. Because we had no a priori reason to think any particular social distancing behavior would be more strongly associated with mental health than another, all indicators were scored equally (one point on the scale).

Work social distancing score was the summed number of positive responses to the following: stayed home from work, was working from home and hadn’t before, or was working or studying from home (all of which were considered to represent the same experience); “left your home and went to work” (reverse-coded), and “cancelled or postponed work or school activities”. Analysis of this variable was limited to those who were employed before the pandemic.

Social distancing from friends/family was the summed number of positive responses to the following: stopped social visits with friends; stopped social visits with family; stopped playdates/visits with children’s friends; gone to friend, neighbor, or relative’s home (reverse-coded); had visitors at your home (reverse-coded); and cancelled or postponed personal or social activities.

Social distancing from public places was the summed number of positive responses to the following: stopped travelling/travel plans; stopped attending worship or religious services; gone out to a bar, club, or place people gather (reverse-coded); attended a gathering with more than 10 people, such as reunion, wedding, funeral, birthday party, concert, or religious service (reverse-coded); avoided public spaces; remained in residence at all times.

A summary measure summed all the above indicators as well as positive responses to “have you been placed in isolation?” and reporting that they lived alone.

On the postpartum questionnaire, participants were also asked whether they had been avoiding: public spaces, gatherings, and crowds; and eating at restaurants.

### Mental health

Timing and sample size for each measure is outlined in Table 1.

*Anxiety* was measured at baseline and follow-up with the Generalized Anxiety Disorder-7 (GAD-7) scale [18]. In both cases, women were asked about their emotional state during the last two weeks. The GAD-7 has been validated in pregnant populations [19].

*Perceived stress* was measured at baseline with the Cohen Perceived Stress scale (PSS) using the10-item version. Questions were asked about the previous month. The PSS has been shown to correlate with depression, social support, and self-esteem in pregnant populations [20].

*Pregnancy-related anxiety* was measured at follow-up with the Perinatal Anxiety Specific Scale (PASS). Questions were asked about the time since the COVID pandemic started. The PASS scale correlates with depression and identifies pregnant persons with significant anxiety [21].

*Pregnancy stress* was measured at baseline using items that asked both about overall feelings of stress and anxiety since the pandemic (3 questions) and feelings of stress and anxiety about the pregnancy specifically (3 questions). This instrument was created to be a snapshot of mental health during the pandemic and has not been validated.

*Depression* was assessed at follow-up and postpartum with the 2-item Patient Health Questionnaire (PHQ-2). This instrument has been validated in pregnant populations and performs reasonably well with a small tendency towards false positives [22].

### Analysis

Each type of social distancing (variable construction provided above) was categorized into quartiles; due to the relatively limited number of responses, the work social distancing variable was categorized into 3 levels. The association between social distancing and mental health factors was examined using ANOVA, and with other covariates using chi-square tests. Covariates were chosen on the basis of being known predictors of mental health during pregnancy (race, marital status, parity, education, and income [categorized as listed in Table 2]) and thought to be potentially associated with social distancing. Participants also reported whether they had a positive COVID-19 test, had been told by a doctor that they had COVID-19, or thought they had been infected with COVID-19. We also examined predictors of social distancing, incorporating all covariates simultaneously into a cumulative logistic model and conducting backward selection until only factors significant at *p* < 0.05 remained. These factors were incorporated as covariates into subsequent models.

**Table 2:** Description of study population

	Baseline sample (*n* = 1037)		Follow-up (*N* = 596)		Postpartum (*N* = 302)	
	N	%	N	%	N	%
Age						
< 25	84	8.2	22	3.7	10	3.3
25- < 30	234	22.8	119	20.1	60	19.9
30- < 35	454	44.3	289	48.7	138	45.9
35 +	254	24.8	163	27.5	93	30.9
Race						
Black	121	11.7	29	4.9	21	7.0
White	872	84.1	546	91.6	270	89.4
Some other race	44	4.2	21	3.5	11	3.6
Marital status						
Single	124	12.0	27	4.5	16	5.3
Married	847	81.8	535	89.8	275	91.1
Domestic partnership/divorced	64	6.2	34	5.7	11	3.6
Educational level						
HS or less	247	23.8	85	14.3	31	10.3
Bachelors’	424	40.9	246	41.3	118	39.1
Masters or higher	366	35.3	265	44.5	153	50.7
Income category						
< 25 K	100	9.8	25	4.2	13	4.3
25 K-100 K	344	33.6	188	31.8	90	29.9
100 K +	580	56.6	378	64.0	198	65.8
Number of children						
0	467	45.0	260	43.6	120	39.7
1	358	34.5	223	37.4	127	42.1
2 +	212	20.4	113	19.0	55	18.2
Trimester						
1	239	23.1	125	21.0	36	11.9
2	384	37.0	249	41.8	125	41.4
3	414	39.9	222	37.3	141	46.7
Month of baseline survey						
May-20	472	45.7	309	51.9	182	60.3
Jun-20	61	5.7	32	5.4	22	7.3
Jul-20	31	3.0	12	2.0	6	2.0
Aug-20	235	22.8	143	24.0	79	26.2
Sep-20	14	1.4	10	1.7	3	1.0
Oct-20	22	2.1	11	1.9	1	0.3
Nov-20	19	1.8	9	1.5	0	0.0
Dec-20	40	3.9	20	3.4	4	1.3
Jan-21	57	5.5	21	3.5	4	1.3
Feb-21	49	4.8	17	2.9	1	0.3
Mar-21	32	3.1	12	2.0	0	0.0

Linear models using mental health factors as continuous outcomes were examined, with social distancing as a categorical major predictor with adjustment for race, marital status, parity, education, and income. Interaction with race and parity was examined in these models using a product term; no interactions were found. For outcomes with both a baseline and measure taken prospectively, a model was also constructed incorporating both the covariates listed above and the baseline mental health levels.

This study was approved by the Institutional Review Board of Woman’s Hospital, and participants provided informed consent during the online survey.

## Results

The study population was largely white (84.1%), married (81.8%), and educated (76.2% with a bachelor’s or higher degree; Table 2). Women were more likely to be lost to follow-up if they were lower-income, lower-education, unmarried, or young (all *p* < 0.05).

Women who were younger, Black, single, had less education, or were lower income reported fewer social distancing behaviors (Table 3). Parous women reported more social distancing from friends and family, but there were not differences across social distancing from work or public places. Those who had previously tested positive for COVID-19 were less likely to observe social distancing. More social distancing behaviors were reported earlier in the pandemic. When these variables were included in a predictive model simultaneously, age, education, parity, and month of survey were the strongest predictors. This held across categories of social distancing (work, family and friends, public places), except that age and parity were not a strong predictor of social distancing from public places (data not shown).

**Table 3:** Social distancing behaviors by covariates

	Age															Race					
	< 25						25–30		30–35		35 +			p		Black		White			p
	N				%		N	%	N	%	N		%			N	%	N	%		
Overall social distancing														< 0.01							< 0.01
1 (lowest quartile)	44				52.4		76	32.5	91	20.0	39		15.4			48	39.7	193	22.1		
2	18				21.4		62	26.5	114	25.1	43		16.9			23	19.0	209	24.0		
3	18				21.4		56	23.9	131	28.9	82		32.3			24	19.8	253	29.0		
4 (highest quartile)	4				4.8		40	17.1	118	26.0	90		35.4			26	21.5	217	24.9		
Work-related														< 0.01							0.08
1 (lowest quartile)	41				56.9		82	37.6	138	31.5	63		25.3			45	42.9	267	31.8		
2/3	15				20.8		77	35.3	130	29.7	69		27.7			27	25.7	252	30.0		
3 (highest quartile)	16				22.2		59	27.1	170	38.8	117		47.0			33	31.4	320	38.1		
Friends and family														< 0.01							< 0.01
1 (lowest quartile)	45				53.6		72	30.8	87	19.2	31		12.2			45	37.2	183	21.0		
2	13				15.5		51	21.8	87	19.2	29		11.4			23	19.0	152	17.4		
3	21				25.0		88	37.6	221	48.7	137		53.9			39	32.2	410	47.0		
4 (highest quartile)	5				6.0		23	9.8	59	13.0	57		22.4			14	11.6	127	14.6		
Public events														< 0.01							< 0.01
1 (lowest quartile)	42				50.0		79	33.8	94	20.7	44		17.3			49	40.5	202	23.2		
2	22				26.2		46	19.7	106	23.4	53		20.9			16	13.2	208	23.9		
3	14				16.7		71	30.3	155	34.1	101		39.8			28	23.1	299	34.3		
4 (highest quartile)	6				7.1		38	16.2	99	21.8	56		22.1			28	23.1	163	18.7		
	Marital status												Education								
	Single					Married			Divorced/widowed			p	HS or less			College		> College			p
	N			%		N		%	N	%			N		%	N	%	N		%	
Overall social distancing												< 0.01									< 0.01
1 (lowest quartile)	53			42.7		186		22.0	15	23.4			101		40.9	101	23.8	53		14.5	
2	27			21.8		193		22.8	17	26.7			59		23.9	100	23.6	79		21.6	
3	27			21.8		245		28.9	18	28.1			55		22.3	126	29.7	109		29.8	
4 (highest quartile)	17			13.7		223		26.3	14	21.9			32		13.0	97	22.9	125		34.2	
Work-related																					< 0.01
1 (lowest quartile)	50			46.7		248		30.4	26	41.9		0.28	104		47.1	136	33.6	86		23.9	
2/3	28			26.2		250		30.7	16	25.8			62		28.1	127	31.4	105		29.2	
3 (highest quartile)	29			27.1		317		38.9	20	32.3			55		24.9	142	35.1	169		46.9	
Friends and family												< 0.01									< 0.01
1 (lowest quartile)	53			42.7		175		20.7	11	17.2			91		36.8	101	23.8	48		13.1	
2	21			16.9		147		17.4	12	18.8			46		18.6	76	17.9	59		16.1	
3	34			27.4		405		47.8	30	46.9			83		33.6	189	44.6	197		53.8	
4 (highest quartile)	16			12.9		120		14.2	11	17.2			27		10.9	58	13.7	62		16.9	
Public events												< 0.01									< 0.01
1 (lowest quartile)	53			42.7		196		23.1	14	21.9			102		41.3	106	25.0	56		15.3	
2	28			22.6		183		21.6	18	28.1			55		22.3	98	23.1	76		20.8	
3	26			21.0		294		34.7	21	32.8			59		23.9	138	32.6	145		39.6	
4 (highest quartile)	17			13.7		174		20.5	11	17.2			31		12.6	82	19.3	89		24.3	
	income												parity								
	< $25 K/year					$25 K-50 K			> $50 K			p	0			1		2 +			p
	N		%			N		%	N	%			N	%		N	%	N		%	
Overall social distancing												< 0.01									< 0.01
1 (lowest quartile)	43		43.0			99		28.9	106	18.3			133	28.5		67	18.7	55		25.9	
2	23		23.0			88		25.6	127	21.9			113	24.2		85	23.7	40		18.9	
3	23		23.0			89		25.7	176	30.3			136	29.1		103	28.8	51		24.1	
4 (highest quartile)	11		11.0			68		19.8	171	29.5			85	18.2		103	28.8	66		31.1	
Work-related												< 0.01									0.34
1 (lowest quartile)	41		45.6			128		39.3	153	27.3			148	33.9		114	32.7	64		32.0	
2/3	25		27.8			99		30.4	168	30.0			132	30.2		107	30.7	55		27.5	
3 (highest quartile)	24		26.7			99		30.4	239	42.7			157	35.9		128	36.7	81		40.5	
Friends and family												< 0.01									< 0.01
1 (lowest quartile)	39		39.0			103		29.9	92	15.9			137	29.3		60	16.8	43		20.3	
2	21		21.0			59		17.2	100	17.2			92	19.7		55	15.4	34		16.0	
3	28		28.0			143		41.6	295	50.9			219	46.9		166	46.4	84		39.6	
4 (highest quartile)	12		12.0			39		11.3	93	16.0			19	4.1		77	21.5	51		24.1	
Public events												< 0.01									0.05
1 (lowest quartile)	44		44.0			105		30.5	110	19.0			137	29.3		73	20.4	54		25.5	
2	20		20.0			74		21.5	133	22.9			100	21.4		86	24.0	43		20.3	
3	25		25.0			92		26.7	224	38.6			149	31.9		125	34.9	68		32.1	
4 (highest quartile)	11		11.0			73		21.2	113	19.5			81	18.9		74	20.7	47		22.2	
Positive covid test																					
Yes									no												
N	%								N			%				p					
																0.01					
8	28.6								86			11.8									
8	28.6								188			25.8									
10	35.7								231			31.7									
2	7.1								223			30.6									
																< 0.01					
14	50.0								178			24.5									
8	28.6								238			32.7									
6	21.4								311			42.8									
																0.11					
8	28.6								104			14.3									
3	10.7								124			17.0									
15	53.6								376			51.7									
2	7.1								124			17.0									
																0.07					
8.0	28.6								103			14.2									
2.0	7.1								176			24.2									
11.0	39.3								274			37.6									
7.0	25.0								175			24.0									

In unadjusted analysis, most of the mental health and stress measures at all time points were associated with social distancing variables (Table 4). The highest levels of mental health symptoms were found in the group observing the most social distancing, while the group observing the least social distancing generally had the lowest level of symptoms. For instance, mean anxiety score in the highest quartile of social distancing was 8.3 (SD 5.6), while in the lowest quartile it was 6.0 (SD 5.0) (*p* < 0.01). Mean perceived stress score in the highest quartile of social distance was 23.0 (SD 3.7), while in the lowest quartile it was 19.7 (SD 5.1) (*p* < 0.01). Mean depression score in the highest quartile of social distance was 3.39 (SD 1.5), while in the lowest quartile it was 2.9 (SD 1.2) (*p* < 0.01). The two exceptions were perceived stress postpartum and pregnancy-related stress. After controlling for covariates, anxiety was associated with overall social distancing, social distancing from friends and family, and social distancing from public events. Associations with work-related social distancing were less strong (Table 5).

**Table 4:** Social distancing by mental health

		Overall social distancing by quartiles				p
		Lowest	2	3	Highest	
Anxiety						
Baseline (cross-sectional; *n* = 1037)	Mean	6.04	6.80	7.85	8.30	< 0.01
	SD	5.04	4.81	5.34	5.64	
Follow-up (prospective, *n* = 596)	Mean	4.91	5.21	6.39	6.65	< 0.01
	SD	4.35	4.28	4.87	4.99	
Follow-up (postpartum, *n* = 302)	Mean	4.58	4.12	6.20	5.82	0.04
	SD	4.66	3.58	5.93	5.23	
General pregnancy stress						
Cross-sectional (*n* = 765)	Mean	12.28	12.94	12.26	13.06	0.30
	SD	5.51	5.09	4.65	5.87	
Overall perceived stress						
Cross-sectional (*n* = 919)	Mean	19.71	21.61	22.42	23.01	< 0.01
	SD	5.07	4.23	4.04	3.65	
Postpartum (*n* = 301)	Mean	14.57	13.70	15.90	15.32	0.26
	SD	6.69	6.14	8.13	6.84	
Depression						
Prospective	Mean	2.89	2.82	3.38	3.39	< 0.01
	SD	1.21	1.04	1.33	1.47	
Postpartum (*n* = 199)	Mean	2.84	2.46	2.96	2.97	0.05
	SD	1.19	0.83	1.44	1.28	
Pregnancy-related anxiety						
Prospective	Mean	20.09	21.38	26.24	25.57	< 0.01
	SD	15.39	15.51	17.34	17.42

**Table 5:** Indicators of social distancing and mental health

	**Anxiety**											
	**Pregnancy**			**Follow-up**						**Postpartum**		
	Beta^a^	SE	p	Beta			SE	p		Beta	SE	p
Overall social distancing												
1 (lowest quartile)	0.00		< 0.01	0.00				< 0.01		0.00		0.01
2	0.87	0.48		0.50			0.67			-0.02	1.05	
3	2.11	0.46		1.91			0.65			2.23	1.00	
4 (highest quartile)	2.61	0.49		2.40			0.67			2.10	1.01	
Work-related^b^												
1 (lowest quartile)	0.00		0.06	0.00				0.14		0.00		0.10
2/3	-0.12	0.45		-0.10			0.55			1.24	0.88	
4 (highest quartile)	0.82	0.44		0.78			0.53			1.80	0.84	
Friends and family												
1 (lowest quartile)	0.00		< 0.01	0.00				< 0.01		0.00		0.03
2	1.68	0.52		1.19			0.72			1.90	1.12	
3	2.65	0.43		2.52			0.58			2.64	0.92	
4 (highest quartile)	2.57	0.58		1.66			0.72			2.85	1.07	
Public events												
1 (lowest quartile)	0.00		< 0.01	0.00				< 0.01		0.00		0.01
2	1.04	0.48		0.72			0.66			0.89	1.01	
3	1.80	0.44		1.33			0.63			0.68	0.92	
4 (highest quartile)	2.48	0.51		2.97			0.70			2.92	0.99	
	**Depression**						**Stress**					
	**Follow-up**			**Postpartum**			**Pregnancy**			**Postpartum**		
	beta	SE	p	beta	SE	p	beta	SE	p	beta	SE	p
Overall social distancing												
1 (lowest quartile)	0.00		< 0.01	0.00		0.02	0.00		< 0.01	0.00		0.13
2	0.01	0.19		-0.33	0.26		1.58	0.44		-0.36	1.48	
3	0.61	0.18		0.20	0.25		2.31	0.43		2.00	1.41	
4 (highest quartile)	0.71	0.19		0.28	0.25		2.74	0.45		1.63	1.42	
Work-related^b^												
1 (lowest quartile)	0.00		0.02	0.00		0.02	0.00		0.01	0.00		0.26
2/3	0.14	0.15		0.09	0.22		0.52	0.37		1.38	1.21	
4 (highest quartile)	0.39	0.15		0.50	0.20		1.08	0.36		1.87	1.15	
Friends and family												
1 (lowest quartile)	0.00		< 0.01	0.00		0.58	0.00		< 0.01	0.00		0.37
2	0.35	0.20		0.06	0.28		1.83	0.48		0.10	1.58	
3	0.68	0.16		0.20	0.23		1.93	0.39		1.75	1.3	
4 (highest quartile)	0.62	0.20		0.34	0.27		2.40	0.50		1.72	1.51	
Public events												
1 (lowest quartile)	0.00		< 0.01	0.00		0.08	0.00		< 0.01	0.00		0.14
2	0.10	0.19		-0.02	0.25		1.18	0.44		1.38	1.42	
3	0.41	0.18		-0.09	0.23		1.75	0.42		0.67	1.3	
4 (highest quartile)	0.56	0.20		0.36	0.24		2.39	0.46		2.78	1.4	
	**Pregnancy stress**						**Pregnancy-related anxiety**					
	**Baseline**						**Follow-up**					
	Beta	SE	p				Beta	SE	p			
Overall social distancing												
1 (lowest quartile)	0.00		0.20				0.00		< 0.01			
2	0.74	0.56					2.20	2.37				
3	0.15	0.54					7.85	2.28				
4 (highest quartile)	1.06	0.58					8.75	2.37				
Work-related^b^												
1 (lowest quartile)	0.00		0.06				0.00		0.18			
2/3	1.11	0.54					0.43	1.93				
4 (highest quartile)	1.08	0.52					2.93	1.84				
Friends and family												
1 (lowest quartile)	0.00		0.52				0.00		< 0.01			
2	-0.46	0.61					7.62	2.52				
3	0.33	0.51					10.03	2.03				
4 (highest quartile)	-0.10	0.69					7.20	2.52				
Public events												
1 (lowest quartile)	0.00		0.55				0.00		< 0.01			
2	0.08	0.56					3.05	2.33				
3	0.30	0.52					5.46	2.24				
4 (highest quartile)	0.83	0.61					10.69	2.47				

^a^all adjusted for age, education, and parity

^b^limited to women who reported employment before pandemic (*n* = 897)

Among the 253 women who had data at baseline and later time points, anxiety was associated with overall social distancing, social distancing from friends and family, and social distancing from public events. Associations were substantially diminished when controlled for baseline levels of anxiety symptoms (Table 6). For instance, the highest quartile of social distancing was associated with a 2.5-point higher anxiety score at follow-up (SD 1.2), but after adjustment this declined to -0.35 (SD 0.8) (corresponding *p*-values = 0.02 and 0.76). Higher social distancing behaviors were associated with higher depression symptoms, but when controlled for baseline levels, depression was lowest in the middle groups of social distancing. Pregnancy-related anxiety was also associated with higher social distancing behaviors, though this was reduced when controlled for baseline anxiety symptoms. Social distancing behaviors were strongly associated with stress cross-sectionally (highest quartile beta 2.74, SD 0.45, *p* < 0.01), but not longitudinally (highest quartile beta 1.63, SD 1.42, *p* = 0.13).

**Table 6:** Indicators of social distancing and mental health, longitudinal (*n* = 253)

	**Anxiety**														
	**Baseline**			**Follow-up**						**Postpartum**					
	Adjusted			Adjusted			Adjusted + baseline			Adjusted			Adjusted + baseline		
	Beta	SE	p	Beta	SE	p	Beta	SE	p	Beta	SE	p	Beta	SE	p
Overall social distancing															
1 (lowest quartile)	0.00		< 0.01	0.00		0.02	0.00		0.76	0.00		< 0.01	0.00		0.29
2	1.95	1.48		0.64	1.28		-0.66	0.82		-0.19	1.44		-0.49	0.91	
3	4.03	1.41		2.52	1.22		-0.16	0.79		2.66	1.37		0.83	0.87	
4 (highest quartile)	4.23	1.42		2.46	1.23		-0.35	0.80		2.41	1.38		0.53	0.88	
Work-related (employed only)															
1 (lowest quartile)			0.45			0.29			0.63			0.48			0.11
2/3	-0.51	1.06		-0.43	0.92		-0.09	0.58		0.57	1.05		1.35	0.74	
4 (highest quartile)	0.54	0.99		0.69	0.86		0.33	0.55		1.14	0.98		1.40	0.70	
Friends and family															
1 (lowest quartile)	0.00		0.01	0.00		0.09	0.00		1.0	0.00		0.10	0.00		0.58
2	2.20	1.49		1.51	1.29		0.04	0.83		0.10	1.46		0.74	0.97	
3	3.76	1.18		2.51	1.02		0.01	0.67		2.05	1.16		0.97	0.81	
4 (highest quartile)	3.43	1.34		2.25	1.16		-0.03	0.75		2.31	1.32		1.27	0.94	
Public events															
1 (lowest quartile)	0.00		< 0.01	0.00		0.03	0.00		0.12	0.00		0.01	0.00		0.14
2	1.76	1.39		-0.64	1.20		-1.82	0.76		1.02	1.35		0.59	0.87	
3	2.83	1.29		0.62	1.12		-1.28	0.72		0.79	1.26		-0.28	0.80	
4 (highest quartile)	4.49	1.36		1.86	1.18		-1.15	0.76		3.23	1.33		1.15	0.87	
	**Depression**														
	**Follow-up**									**Postpartum**					
	Adjusted									Adjusted			Adjusted + baseline		
	Beta			SE		p				Beta	SE	p	Beta	SE	p
Overall social distancing															
1 (lowest quartile)	0.00					< 0.01				0.00		< 0.01	0.00		0.01
2	0.02			0.33						-0.81	0.35		-0.95	0.31	
3	0.69			0.32						-0.10	0.33		-0.52	0.30	
4 (highest quartile)	0.53			0.32						-0.06	0.33		-0.43	0.30	
Work-related (employed only)															
1 (lowest quartile)	0.00					0.15						0.06			0.28
2/3	0.01			0.24						-0.09	0.25		-0.10	0.21	
4 (highest quartile)	0.33			0.23						0.35	0.24		0.17	0.20	
Friends and family															
1 (lowest quartile)	0.00					0.21				0.00		0.24	0.00		0.03
2	0.40			0.34						-0.47	0.35		-0.93	0.32	
3	0.56			0.27						0.01	0.28		-0.36	0.25	
4 (highest quartile)	0.55			0.31						0.13	0.32		-0.33	0.29	
Public events															
1 (lowest quartile)	0.00					0.16				0.00		0.13	0.00		0.10
2	-0.03			0.32						-0.36	0.33		-0.45	0.31	
3	0.32			0.30						-0.35	0.31		-0.55	0.29	
4 (highest quartile)	0.41			0.31						0.06	0.32		-0.22	0.31	
	**Pregnancy-related anxiety**														
	**Follow-up**														
	Adjusted									**Adjusted + baseline**					
	Beta			SE		p				Beta	SE	p			
Overall social distancing															
1 (lowest quartile)	0.00					< 0.01				0.00		0.24			
2	2.20			2.37						-2.24	1.82				
3	7.85			2.28						0.42	1.78				
4 (highest quartile)	8.75			2.37						-0.14	1.85				
Work-related (employed only)															
1 (lowest quartile)	0.00					0.18				0.00		0.87			
2/3	0.43			1.93						-0.09	1.43				
4 (highest quartile)	2.93			1.84						0.52	1.37				
Friends and family															
1 (lowest quartile)	0.00					< 0.01				0.00		0.41			
2	7.62			2.52						3.06	1.94				
3	10.03			2.03						2.14	1.60				
4 (highest quartile)	7.20			2.52						1.22	1.95				
Public events															
1 (lowest quartile)	0.00					< .0001				0.00		0.17			
2	3.05			2.33						-1.45	1.79				
3	5.46			2.24						-0.50	1.73				
4 (highest quartile)	10.69			2.47						1.88	1.93				
	**Perceived stress**														
	**Baseline**									**Postpartum**					
	Adjusted									Adjusted			Adjusted + baseline		
	Beta			SE		p				Beta	SE	p	Beta	SE	p
Overall social distancing															
1 (lowest quartile)	0.00					0.01				0.00		0.17	0.00		0.31
2	1.70			0.94						-0.88	1.74		-2.02	1.64	
3	2.41			0.91						1.49	1.68		-0.12	1.59	
4 (highest quartile)	3.07			0.92						1.16	1.70		-0.89	1.61	
Work-related (employed only)															
1 (lowest quartile)	0.00					0.28				0.00		0.43	0.00		0.71
2/3	0.33			0.72						0.98	1.26		0.76	1.17	
4 (highest quartile)	0.99			0.68						1.56	1.20		0.90	1.11	
Friends and family															
1 (lowest quartile)	0.00					0.02				0.00			0.00		0.73
2	2.40			0.96						0.52	1.76	0.48	-1.09	1.66	
3	2.54			0.81						1.90	1.49		0.20	1.41	
4 (highest quartile)	2.30			0.91						1.82	1.69		0.28	1.59	
Public events															
1 (lowest quartile)	0.00					< 0.01				0.00		0.18	0.00		0.31
2	0.29			0.88						1.05	1.63		0.85	1.52	
3	1.58			0.82						0.38	1.52		-0.68	1.43	
4 (highest quartile)	2.39			0.87						2.50	1.61		0.89	1.52	

Women in the top category of isolation from public places at baseline were likely to report avoiding both crowds and eating in restaurants (92%) postpartum compared to those in the bottom category (although 70% still reported avoiding these, *p* < 0.01). Examining the correlations between the isolation and anxiety at the two time points, social distancing at baseline was a statistically stronger predictor of anxiety postpartum (*p* = 0.01) than social distancing postpartum (*p* = 0.10), although the beta coefficient was similar in size for the top category in both groups (beta = 2.6). However, once baseline anxiety was controlled for, neither social distancing at baseline nor postpartum was associated with postpartum anxiety.

## Discussion

Our goal in this analysis was to better understand the association between various types of social distancing with mental health and perceived stress. We found that overall social distancing, social distancing from friends and family, and social distancing from public places were associated with anxiety. Social distancing from work had a less powerful impact. This is consistent with other research that revealed the social support garnered by friends and family can be extremely beneficial in coping with stressful situations [9]. The participants reported social distancing most strongly at the beginning of the pandemic, which is unsurprising given that restrictions were also most extreme at this time. In an online, worldwide survey of pregnant and postpartum women, distancing was associated with symptoms of PTSD and perhaps higher depression and loneliness, while no associations were seen with changes in travel [3].

We hypothesized that social distancing would lead to worse mental health, and the data provide some support for this hypothesis. However, our results in some ways are more consistent with the reverse association: people who are more anxious are probably likely to adhere to public health recommendations more closely. When results were adjusted for baseline levels of mental health, many associations between social distancing and mental health were reduced or disappeared. This is broadly consistent with a Norwegian study which found that pregnant women who reported frequent voluntary isolation as well as those who had pre-existing mental health conditions were more likely to have postpartum anxiety or depression [23], as well as a Utah study indicating that emotional dysregulation was a stronger predictor of the progression of mental health among pregnant individuals than community levels of COVID-19 cases [24]. However, it should be noted that for some measures, the time frame between measures may have overlapped – for instance, questions on anxiety cover the last two weeks. If women took the follow-up survey within two weeks of this measure, the adjustment for baseline symptoms may be too conservative. The study baseline was well into the pandemic and mental health symptoms at that point may have been caused by the stressors of the previous months, so this is not a perfect control for propensity to anxiety. Similarly, those who were younger, and potentially less worried about contracting the disease, were less likely to report social distancing. Those who had previously tested positive for COVID-19 were also less likely to report social distancing; either behaviors like lack of social distancing might have put them at higher risk, or they might feel unlikely to get the disease again. We asked about social distancing only at baseline, so people’s behaviors may have changed, especially as restrictions were lifted later in the study time frame; the mental health impacts of social distancing may have been reduced in the later parts of the study. Other constraints also affected the degree of reported social distancing behaviors. Some types of employment do not allow for remote work, while participants who were unemployed or had no other children would not have experienced social distancing from work or children’s activities.

Surveillance of pregnancy can provide information about the population at large, especially for infectious disease [25]. We were able to recruit a large sample rapidly. Validated instruments were used for mental health, but not for social distancing (such validated instruments likely did not exist at this point), but no mental health diagnoses were conducted. However, although recruitment was general and Woman’s Hospital attends to a wide cross-section of the population, the included sample is largely white, educated, and of a middle to high socioeconomic status. This limits generalizability, as such women were less financially affected and were likely more able to isolate, and they may not have had jobs that required in-person work. The high degree of loss to follow-up means the prospective analyses are limited in sample size and may be an even more selected population. The lack of financial incentives or personal contact with a recruiter likely contributed to the high loss to follow-up. The time frame of the study (April 2020-March 2021) means that it was conducted in the earlier stages of the pandemic, while the state was operating under at least some limitations, and before vaccinations were available, so we do not know how these factors or later variation in local public health restrictions may have changed the pandemic impacts on mental health. In addition, we have limited information on how social distancing behaviors may have changed over time, as these questions were asked primarily at baseline.

Social distancing during pregnancy is associated with several factors, in many cases bidirectionally with mental health (Figure S1). Women with multiple children may have more financial hardships, stress, anxiety, and domestic responsibilities, but may also feel less lonely than women without children or partners. We did not ask about measures people took to mitigate isolation, such as virtual social groups or outside gatherings. Such measures may be important for improving mental health [26], though organizing them may be particularly difficult at a stressful time. Women in unstable or unhealthy relationships are at risk for domestic violence, something we did not measure in this study. Several studies suggest that domestic violence has increased during the pandemic, and that social isolation is a contributing factor [27]. Structural factors such as immigration status may leave women vulnerable, though in this study we did not see relationships with race or other factors that might indicate greater risk for exposure.

## Conclusions

Maternal perinatal anxiety and depression have been associated with poorer child social-emotional, cognitive, language, and behavioral development, and these effects extend into adolescence [28]. For this reason, the finding that adherence to public health restrictions may be associated with more anxiety, either as a cause or effect, has worrisome implications for the long-term effects of the pandemic. Gender disparities have become even more grave during the pandemic, especially for single and working mothers [29]. However, anxiety and stress can be mitigated, and interventions are available [30]. A study of low-income mothers concluded that peer support groups and home visits improved both the mother's mental health and the child's health outcomes [31], a conclusion that has been replicated in other studies, identifying the positive impact of increased social support during pregnancy [32]. Primary health care providers have been advocating that practitioners engage in social prescribing with their patients, encouraging them to participate in community activities that adhere to current public health precautions [33].

Such interventions would need modifying to address pandemic restrictions. Due to the public health imperatives towards both maternal and child health, policy recommendations encourage the use of technology by hospitals and clinics to combat some of these concerns [34]. Obstetricians recommend using antenatal care visits to access the mental health of the patient, and ensure that they have the support they need [35]. While those with diagnosed mental health conditions are of particular concern, under pandemic conditions, anxiety may be a disorder when a “sick population” rather than “sick individuals” (to use Rose’s formulation [36]) should be targeted for interventions. Innovative models for connection, such as virtual support groups or low-risk outdoor activities, may need to be provided in an easy-to-access manner. Communities have become more isolated in recent decades [37], and loneliness and depressive symptoms act in concert to further degrade overall well-being [38]. While every subset of the population has felt the emotional and psychological impact of the COVID-19 pandemic, special attention must be paid to vulnerable populations, with pregnant women being of paramount concern [39].

## Supplementary Information

**Additional file 1: Figure S1.** Relationships among social risk factors, mental health, and social distancing during pregnancy.

## Data Availability

The datasets used during the current study are available from the corresponding author on reasonable request and appropriate ethical approvals.
