# Supplementary material for: Social distancing and mental health among pregnant women during the coronavirus pandemic
Source: BMC Womens Health. 2023 Apr 20;23:189. doi: 10.1186/s12905-023-02335-x (PMC10117246; doi:10.1186/s12905-023-02335-x)

Figure S1. Relationships among social risk factors, mental health, and social distancing during pregnancy


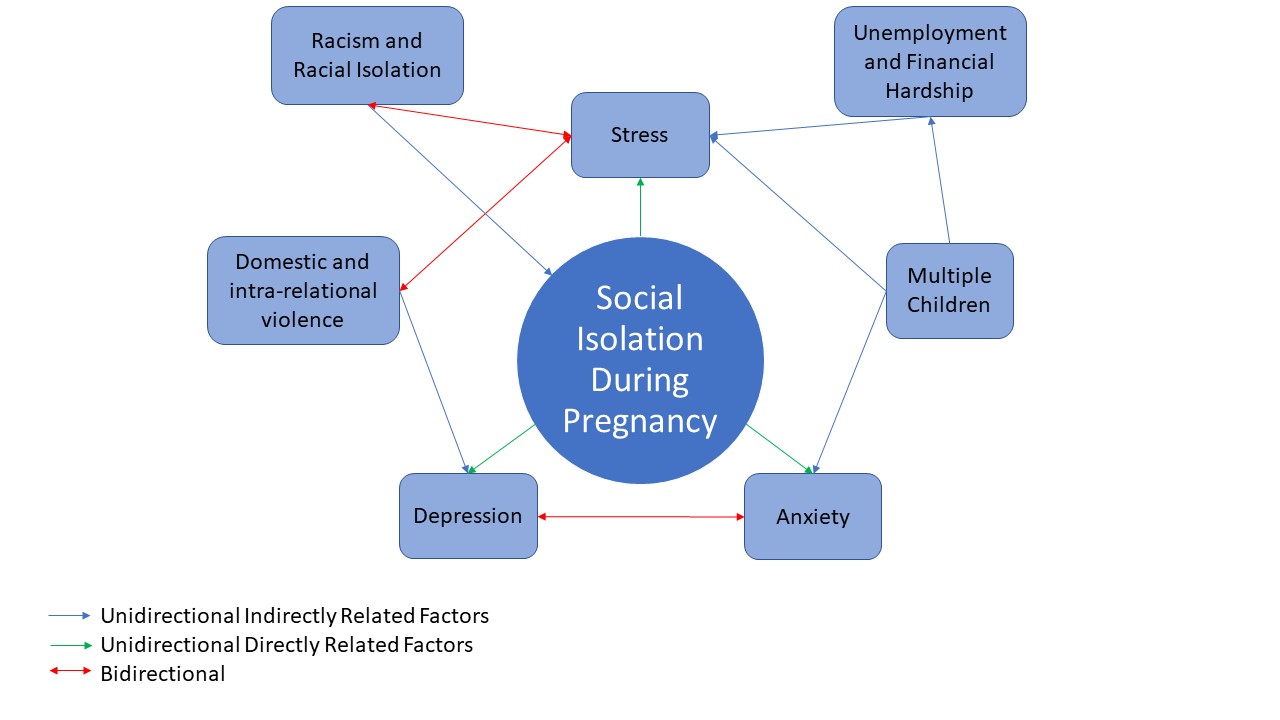

Supplement: Supplementary file 1 — Additional file 1: Figure S1. Relationships among social risk factors, mental health, and social distancing during pregnancy. [file 12905_2023_2335_MOESM1_ESM.docx]
